# Study on Differences in the Pathology, T Cell Subsets and Gene Expression in Susceptible and Non-Susceptible Hosts Infected with *Schistosoma japonicum*

**DOI:** 10.1371/journal.pone.0013494

**Published:** 2010-10-18

**Authors:** Weibin Jiang, Yang Hong, Jinbiao Peng, Zhiqiang Fu, Xingang Feng, Jinming Liu, Yaojun Shi, Jiaojiao Lin

**Affiliations:** 1 Shanghai Veterinary Research Institute, Chinese Academy of Agricultural Sciences, Key Laboratory of Animal Parasitology, Ministry of Agriculture of China, Shanghai, People's Republic of China; 2 East China Normal University, School of Life Science, Shanghai, People's Republic of China; The George Washington University Medical Center, United States of America

## Abstract

More than 40 kinds of mammals in China are known to be naturally infected with *Schistosoma japonicum* (*S. japonicum*); *Microtus fortis* (*M. fortis*), a species of vole, is the only mammal in which the schistosomes cannot mature or cause significant pathogenic changes. In the current study, we compared the differences in pathology by Hematoxylin-eosin staining and in changes in the T cell subsets with flow cytometry as well as gene expression using genome oligonucleotide microarrays in the lung and liver, before challenge and 10 days post-infection with schistosomes in a *S. japonicum*-susceptible mouse model of infection, a non-susceptible rat model and the non-permissive host, *M. fortis*. The results demonstrated that *S. japonicum* promoted a more intensive immune response and more pathological lesions in *M. fortis* and rats than in mice. Hematoxylin-eosin staining revealed that the immune effector cells involved were mainly eosinophilic granulocytes supplemented with heterophilic granulocytes and macrophages. The analysis of splenic T cell subsets showed that CD4^+^ T cell subsets and the CD4^+^/CD8^+^ ratio were increased, while the CD8^+^ T cell subsets decreased remarkably in rats; whereas the CD8^+^ T cell subsets were increased, but the CD4^+^/CD8^+^ ratio was decreased significantly in mice. The analysis of the pattern of gene expression suggested that some immune-associated genes and apoptosis-inducing genes up-regulated, while some development-associated genes were down-regulated in the infected *M. fortis* compared to the uninfected controls; the three different hosts have different response mechanisms to schistosome infection. The results of this study will be helpful for identifying the key molecules in the immune response to *S. japonicum* in *M. fortis* and for understanding more about the underlying mechanism of the response, as well as for elucidating the interaction between *S. japonicum* and its hosts.

## Introduction

Schistosomiasis japonica, which is widely distributed in many parts of the tropics, is one of the most serious zoonotic diseases caused by a parasite [1]–[3]. There are about 46 species of mammals known to carry a natural infection with *S. japonicum* and *Microtus fortis* (*M. fortis*) are the only mammals in which it has been experimentally confirmed to be non-permissive to schistosome infection [4], [5]. In the early 1960s, more than ten thousand wild *M. fortis* living in the areas where schistosomiasis is heavily endemic were examined by Chinese parasitologists; no adult worms or *S. japonicum* eggs were found in any of the animals [6]. Previous studies had revealed that the susceptibility of different types of hosts was varied. Liu et al. reported that the susceptibility to *S. japonicum* was different among the five species of field rats commonly found in the endemic areas; the more susceptible to less susceptible species are: *Rattus eloquens*, *R. hainanicus*, *R. flavipectus*, *R. rattoides* and *R. norvegicus* [7]. The lung of the definitive host is the first stop that the schistosomule migrate to and most of the worms are consumed in this organ of the infected *M. fortis* [8]. The liver is the site of schistosomes maturation. In order to understand more about the mechanisms underlying the non-permissiveness of *M. fortis* and the interaction between the hosts and the schistosomes, the differences between the lungs and liver of BALB/c mice (susceptible host for *S. japonicum*), Wistar rats (non-susceptible host) and *M. fortis* (non-permissive host) infected or uninfected with *S. japonicum* were analyzed and compared on the tissular, cellular and molecular levels.

## Results

### Observation of the histopathology in the lungs and liver of different hosts infected with *S. japonicum*

**Figure 1:**
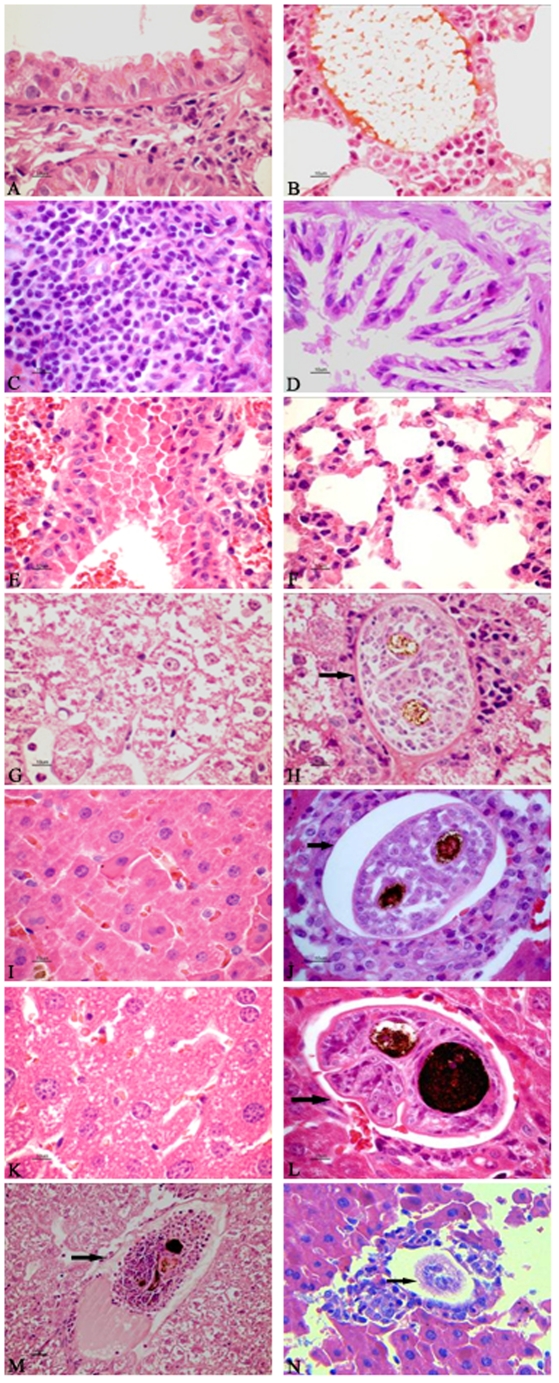
Micrographs of HE staining of the tissue sections prepared from *Mf*, rats and mice. (a–f) Lung tissue from (a) uninfected *Mf*, (b) infected *Mf*, (c) uninfected rats, (d) infected rats, (e) uninfected mice and (f) infected mice. (g–l) Liver tissue from (g) uninfected *Mf*, (h) infected *Mf*, (i) uninfected rats, (j) infected rats, (k) uninfected mice and (l) infected mice. (m) Schistosomulum killed in the liver of infected *Mf* and (n) infected rats. The arrowheads indicate the cross section of *S. japonicum* and two deep colored rounds are its intestinal canal. (a–1)×400 (m) and (n)×200.

In the lungs of *M. fortis*, infected for 10 days with *S. japonicum*, hemorrhages were visible and, in the liver, there were many worm-granulomas detected, schistosomula was showing impaired development surrounded by inflammatory cells [9]. Neither of these phenomenon were seen in the mice or rats infected with *S. japonicum*. Compared with the control groups (Figure 1a), a large number of inflammatory cells, including eosinophils and lymphocytes, exuded around the blood vessels and bronchi of the lungs in the *S. japonicum*-infected *M. fortis* after 10 d. Rat lungs displayed mild pathologic damage and contained some inflammatory cells around the blood vessels and the bronchi. Though inflammation in the lungs of mice was not as serious as that of the lungs from the *M. fortis* or rats, some erythrocytes had infiltrated the lungs and the alveolar walls were edematous (Figure 1b, d, f).

The histological sections of the liver from the control animals from each species demonstrated that the structural integrity of the hepatic lobules was intact, there was actinomorphous distribution of the hepatic cord centered by central veins, polygonal hepatocytes and no edema (Figure 1g, i, k). Ten days after *M. fortis* was infected with *S. japonicum*, some hepatocytes displayed mild swelling, there were a large number of eosinophils and few neutrophils, macrophages and lymphocytes infiltrating the liver and surrounding the schistosomulum. Some of the schistosomulum had been killed. The inflammatory reaction was also severe in the livers of the rats, there were a number of inflammatory cells aggregated around the schistosomulum; however, this was not so obvious in the livers from the infected mice, in which some of the hepatocytes were involved and were shrinking and degenerating (Figure 1h, j, l, m, n).

### Comparison of rat and mouse splenic T cell subsets between infected and uninfected animals

Double-color analysis of cells was performed using FCM. The proportion of splenic CD4^+^ and CD8^+^ T cells was measured (Table 1). Rats infected with *S. japonicum* displayed a significant increase in the percent of CD4^+^ T cells and CD4^+^/CD8^+^ T cell ratio, and a significant reduction in the percent of CD8^+^ T cells when compared to uninfected rats. In mice, although no differences in the CD4^+^ T cell subsets were noted, the percentage of CD8^+^ T cell was significantly higher and CD4^+^/CD8^+^ T cell ratio was significantly lower in the infected group compared to the uninfected group. Few CD4^+^ or CD8^+^ T cells were detected in the spleen of the *M. fortis* by FCM.

**Table 1:** Results of CD4^+^ and CD8^+^ T cell ratio of spleen (Mean ± Std).

	CD4^+^ (%)	CD8^+^ (%)	CD4^+^/CD8^+^ ratio
***Rats***			
Uninfected	24.43±2.93	35.14±1.10	0.70±0.16
Infected	31.19±1.77*	21.15±3.25*	1.48±0.11*
***Mice***			
Uninfected	18.77±2.00	9.38±1.46	2.01±0.14
Infected	21.26±1.90	12.30±0.61*	1.74±0.24*

*p<0.05, as compared to the corresponding uninfected group.

### Detection of RNA quality

It is important to identify any differences in the quality of the isolated total RNA, since this can compromise the measurement of gene expression. The ratio of A260/280 of all tested RNA were in the range of 1.8–2.0, we ensured that all the total RNA samples were of high quality and quantity with minimal degradation, as recommended by Bustin [10].

### Comparison of the gene expression in the lungs and liver of uninfected animals and animals infected with *S. japonicum* for 10 days

**Figure 2:**
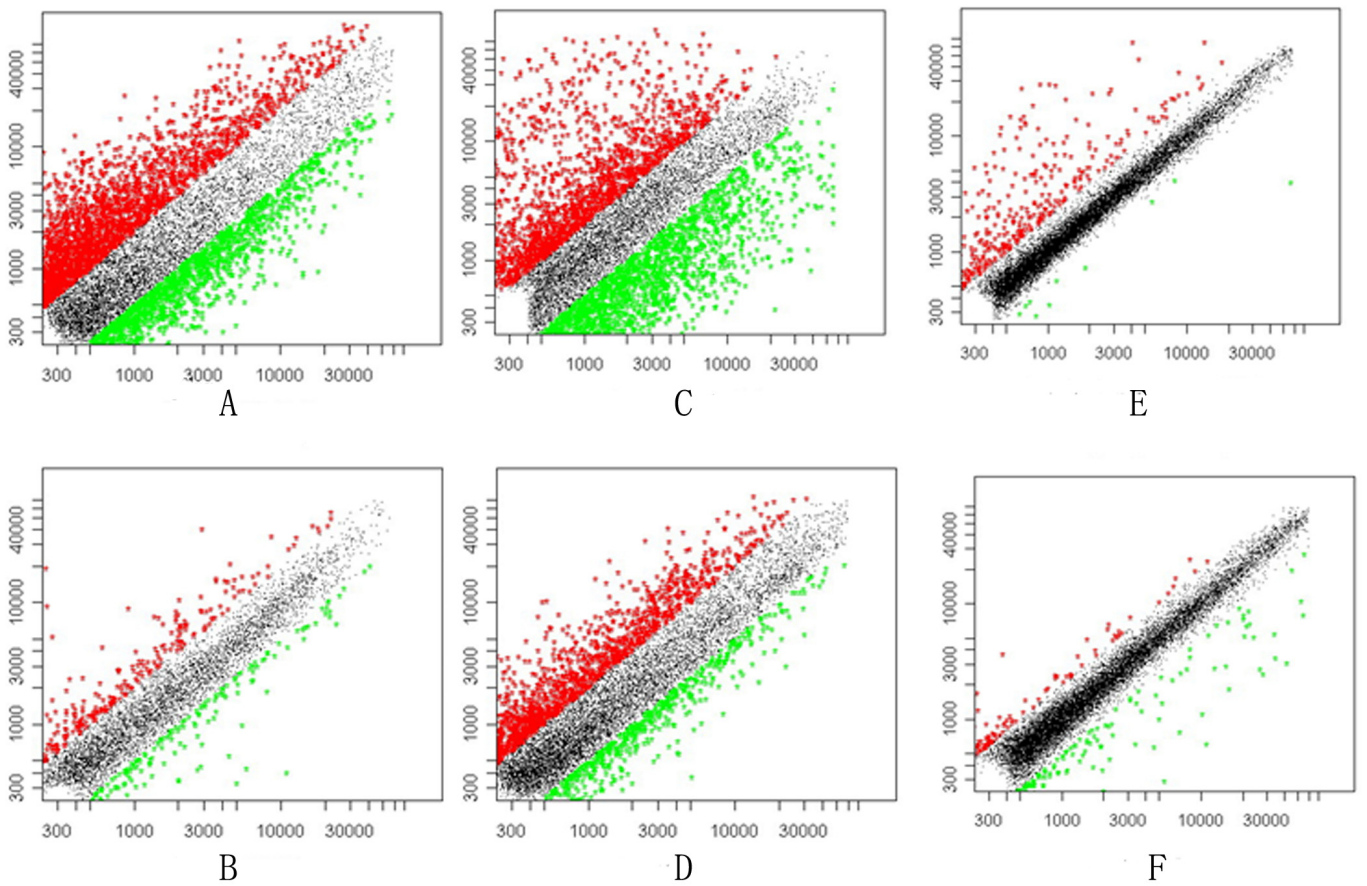
Scatter plot of microarrays. The Gene expression were measured by microarrays from (A) *M. fortis* lungs; (B) *M. fortis* livers; (C) rat lungs; (D) rats livers; (E) mice lungs and (F) mice livers.

**Figure 3:**
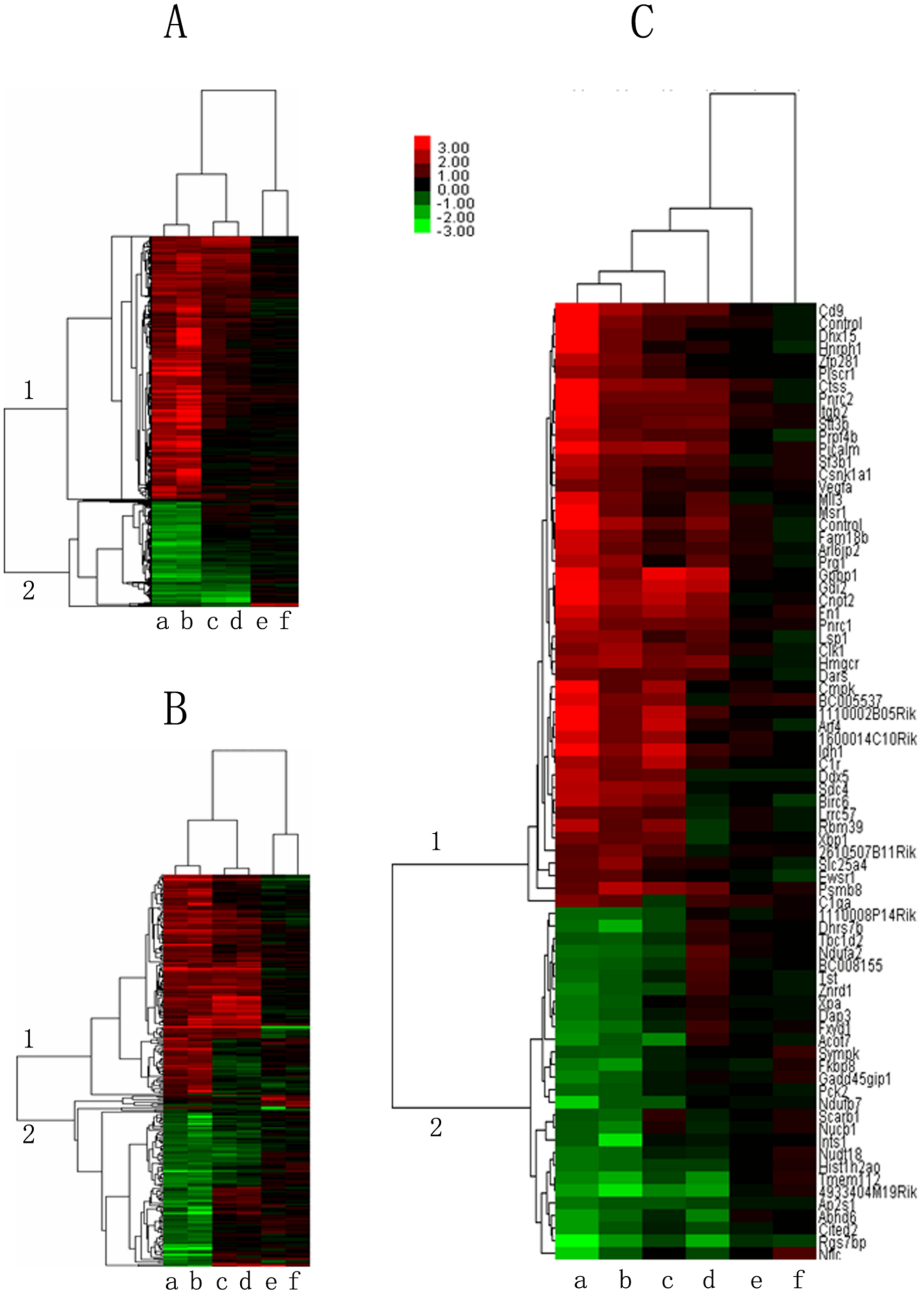
Gene expression analyses of *M. fortis*, rats and mice. (A) Dendrogram displaying the hierarchical clustering of the genes in the lungs of the three species according to the hybridization signals of 1334 probe sets detected (a–b, *M. fortis*; c–d, rats; e–f, mice). Columns correspond to the different lung samples in the same order as in the dendrogram. Rows represent the individual probe sets. For each probe set, red, green, and black indicate increased, decreased, and equal hybridization levels relative to the median, respectively. Hierarchical cluster analyses classified the probe sets into two different groups related to their species-specific hybridization pattern. Clusters 1 and 2 contained 1425 probe sets (1334 genes) with an *M. fortis*-specific hybridization pattern, and they include increased (cluster 1, 895 genes) and decreased (cluster 2, 439 genes) signals in *M. fortis* compared with rats and mice. (B) Hierarchy cluster analysis showing that 282 probe sets (265 genes) were identified as differentially regulated, which were increased (cluster 1, 155 genes) and decreased (cluster 2, 110 genes) in the liver of *M. fortis*, were relatively different in the rat liver and nearly fixed in the mouse. (C) Hierarchy cluster analyses showing gene changes in the liver and lung. The expression of 46 genes was increased (cluster 1)and 28 genes were decreased (cluster 2) in *M. fortis*, were relatively different in rats and were nearly fixed in mouse. (g, *M. fortis* lungs; h, *M. fortis* liver; i, rat lungs; j, rat liver; k, mouse lungs; l, mouse liver).

**Figure 4:**
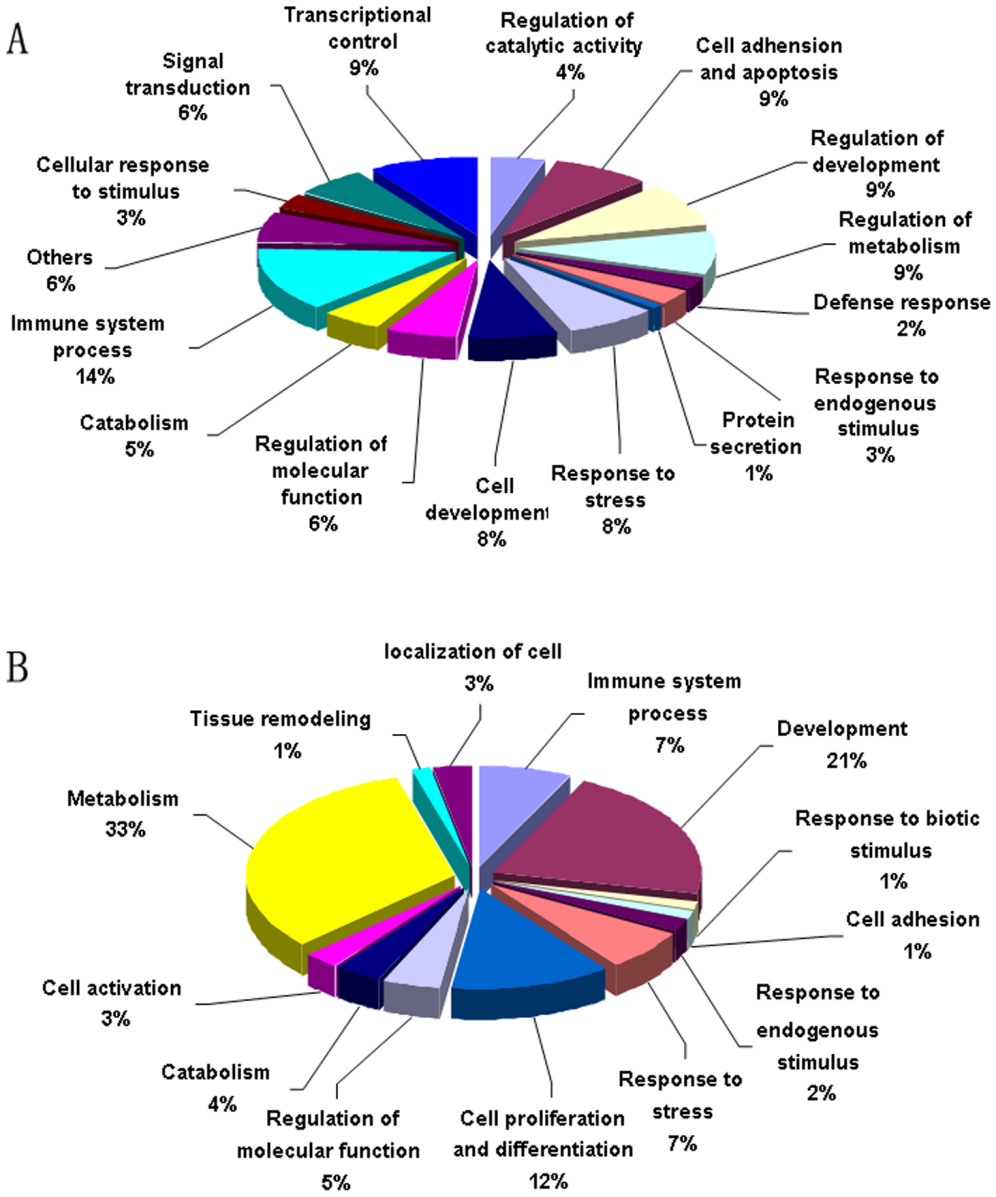
Distribution of major gene ontology categories of genes with differential expression in the three species. (A) In the lungs, the informative sequences can mainly be grouped into 16 groups. (B) in the livers, This analysis revealed that around 2/3 genes are related with metabolism, development, cell proliferation and differentiation.

The differences in gene expression in the lungs and liver of uninfected and infected animals were analyzed using genome oligonucleotide microarray (see Table 2). As shown in Figure 2, scatter plots of the chip data demonstrated that the differences in the genes expressed in the uninfected compared to the infected animals were greater among groups of *M. fortis* and rats than among the infected and uninfected mice; expression differences were also more robust in the lungs than in the liver. The homologous genes that were differentially expressed in the lungs and liver of three species were further analyzed by hierarchical cluster and reviewed using Treeview. These results demonstrated that most of these genes were significantly changed in the *M. fortis*, mildly altered in rats and there was almost no difference among the mice. Gene expression analyses identified 1334 genes with clear differential expression in the lungs (Figure 3a and Table 3). Among the sequences, 650 sequences had their GO (gene ontology) functional annotations and the GO analysis was performed through DAVID online tool [11]. Examining the GO tree, the informative sequences can mainly be grouped into 16 groups (Figure 4a). This analysis revealed that the predicted biological functions of these genes were mainly related to immune system process (14%), transcriptional control (9%), cell adhesion and apoptosis (9%), etc. In the liver, 265 genes were found to have differential expression (Figure 3b) and 183 sequences had their GO functional annotations. This analysis revealed that around 2/3 genes are related with metabolism (33%), development (21%), cell proliferation and differentiation (12%) (Figure 4b). We then focused on analyzing the genes that were expressed similarly in the lungs and liver. Cluster analyses showed that 46 genes were significantly up-regulated in *M. fortis* while there was no change in these genes in mice after challenge with *S. japonicum* for 10 days (Figure 3c). The prediction of biological function revealed that these genes were mainly linked to signal transduction (*Msr1 and Pnrc1*), gene transcriptional regulation (*Cnot2*, *Znrd1*, *Xbp1 and Gpbp1*) and the immune response (*C1qa*, *C1ra*, *C1rb*, *Psmb8 and Itgb2*). Another 28 genes were significantly down-regulated in *M. fortis* but unchanged in mice, their predicted biological functions were mainly related to protein binding (*Nudt18 and Fkbp8*) and transferring enzyme activation (*Dap3 and Nudt18*) (Table S1). Table S2 shows all the changed expression genes in the three species.

**Table 2:** Comparison and analysis of gene expression profiles in the lungs and liver of animals uninfected and infected with *S. japonicum*.

group (infected/uninfected)	clones detected	percentage detected	up-regulated probe sets	up-regulated genes (SR≥2)	up-regulated genes (SR≥4)	up-regulated probe sets	down-regulated genes (SR≤0.5)	down-regulated genes (SR≤0.25)
***Lungs***								
*M. fortis*	10600	39%	2678	1858	990	1478	1049	212
Rat	10528	39%	1980	1582	872	2306	1719	1068
Mouse	14208	40%	364	290	140	16	13	3
***Liver***								
*M. fortis*	5412	20%	330	248	51	190	172	24
Rat	10462	38%	1655	1313	381	540	426	48
Mouse	13654	38%	111	95	11	179	109	38

**Table 3:** The expression changes in genes in the lungs and liver of *M. fortis* and of mice.

	*Lungs*		*Liver*		Homologous b/w lungs & liver	
Expression changes b/w species	probe sets	genes	probe sets	genes	probe sets	genes
Upregulated in *M. fortis*, unchanged in mice	952	**895**	167	**155**	50	**46**
Unchanged in *M. fortis*, downregulated in mice	2	2	7	6	0	0
Upregulated in *M. fortis*, downregulated in mice	1	1	5	5	0	0
Upregulated in mice, unchanged in *M. fortis*	55	52	18	17	0	0
Unchanged in mice, downregulated in *M. fortis*	473	**439**	115	**110**	29	**28**
Upregulated in mice, downregulated in *M. fortis*	19	17	2	2	0	0

### Analysis of the immune-, development- and apoptosis-associated genes

Further analyses of the differential expression of genes revealed that some immune-associated genes and apoptosis-promoting genes in *M. fortis* infected with *S. japonicum* for 10 days were up-regulated, including non-specific immune-related genes (*C1qa*, *C8a* in lungs and *Irf7* in liver) and specific immune-related genes (*Cgr1*, *Cgr3* in lungs and *Cd74* in livers), as well as some apoptosis-promoting genes (*Pdcd6*, *Casp7* and *Tyk2*). At the same time, a few development-associated genes (*Thra*, *Thrsp*, *Hsd11b1* in lungs and *Igf1* in livers) were down-regulated in *M. fortis*.

### Conjecture and analysis of the mechanisms against schistosome infection in the three host species

Further analyses of gene expression suggested that the lungs and liver of the three different hosts may have different response mechanisms to schistosome infection, such as signaling through Jak-STAT, VEGF, or Notch, as well as the FcεRI signaling pathways in *M. fortis*, the complement cascade in rats and interaction among a variety of cytokines (mainly chemokines and TNF) and Ca2 ^+^ signaling pathways in mice.

### Confirmation with Real-time RT-PCR

**Figure 5:**
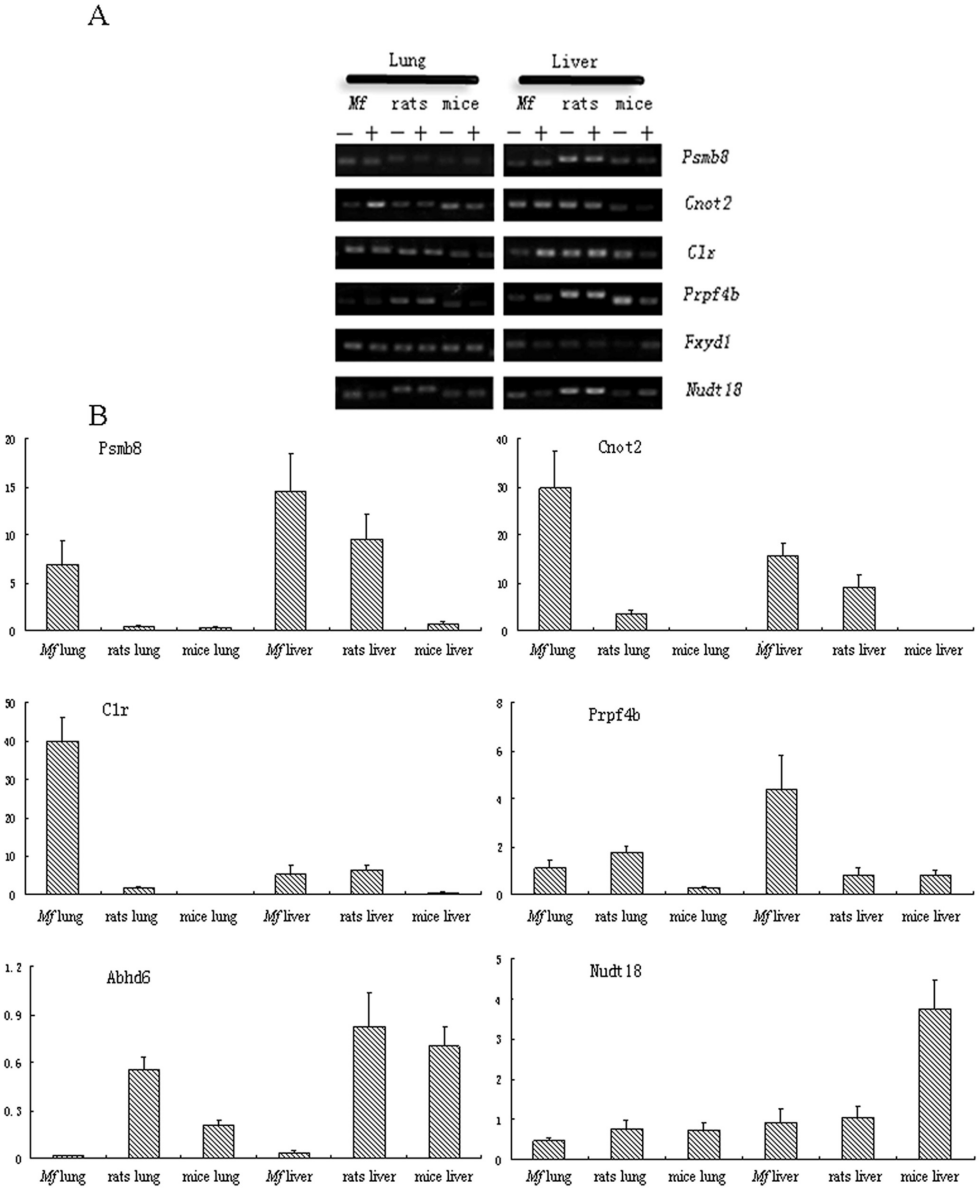
Result of real-time RT-PCR confirmation of gene chip data. (A) The mRNA level was measured using RT-PCR and the same sample utilized for the microarray analyses. The amplification reactions were cycled for 30 cycles. −, Uninfected group; +, Infected group. (B) The same reactions were performed in a real-time system. These six histograms presented the expression data for six genes (Psmb8, Cnot2, Clr, Prpf4b, Abhd6 and Nudt18) from 74 genes with differential expression in the lungs and livers of the animals separately.

Real-time RT-PCR was used to quantitate the expression of six genes from 74 genes with differential expression in the lungs and livers of the three rodent species (Table S1). Three PCRs with *Psmb8* in rat lung, *Cnot2* in rat liver and *Prpf4b* in *M.fortis* lung produced products that were relatively under-expressed compared with the microarray data while a PCR amplification of *Abhd6* in rat lung was relatively over-expressed. The remaining amplicons obtained by Real-time RT-PCR all correlated well with the fold changes generated from the microarray analysis (Figure 5).

## Discussion

Mice are susceptible to *S. japonicum* while rats are non-susceptible and *M. fortis* is a non-permissive host. After infection with cercarie under laboratory conditions, the developmental rate of *S. japonicum* in mice and rats was around 65% and 15%, respectively. Most of schistosomula were dead in *M. fortis* by 15 days post-infection. Previous studies have demonstrated that no pathological changes are seen in the lungs or liver of infected *M. fortis* compared to uninfected controls as far out as 42 days post-infection [8], [12]. Since the three types of hosts have different genetic, physiological and biochemical characteristics, they provide distinct living environments for the schistosomes that directly and indirectly influence the development and survival of the worms. The current paper analyzed and compared the differences between the infected and uninfected animals at the tissue, cell and gene expression level among the three species in order to understand more regarding the mechanisms of the development of schistosomes and of the anti-schistosome response in *M. fortis*.

Studies evaluating the histiocytic reaction against the trematodes, *Paragonimus Westermani*, in non-susceptible hosts demonstrated that the inflammatory cells surrounding the organism included a large number of eosinophils, as well as some neutrophils, macrophages and mast cells, in the muscle tissue of challenged rats [13]. He et al. examined the dermal reaction in eight host species that were primarily infected with *S. japonicum* and reported that the schistosomulum in the skin of birds and other non-susceptible hosts were surrounded by an eosinophil infiltrate predominantly, while there was only a light reaction, mediated by a few infiltrating neutrophils, in the skin of susceptible hosts [14]. Our study revealed that the lungs and liver of *M. fortis*, rats and mice display different characteristics when they were challenged with *S. japonicum* for 10 days. In *M. fortis*, the lungs contain a large number of eosinophils and lymphocytes effused around bronchial and blood vessels and the bronchial mucosa and surrounding tissue was edematous. In the liver of *M.fortis*, schistosomulum were surrounded by an inflammatory infiltrate composted mainly of eosinophils and a small number of neutrophils, macrophages and other cells. Liu et al. established a test for evaluating the killing effect of the immune response in *M. fortis* to schistosomulum in vitro using antibody-dependent cell-mediated cytotoxicity (ADCC) [15]. They found that, after incubating macrophages and eosinophils from *M. fortis* or from mice with *S. japonicum*-infected sera or normal sera for 6 h, there were many more cells binding to the schistosomulum in the cultures containing granulocytes from *M. fortis* than those containing mouse-derived cells. The results indicated that macrophages and eosinophils from *M. fortis* were capable of more strongly adhering to schistosomulum naturally, and that eosinophils may be one of the important effector cells for killing schistosomes in *M. fortis* and other non-susceptible hosts. *M. fortis* and rats, both of which are resistant hosts to *S. japonicum*, developed a stronger immune response and more severe pathological lesion in response to the schistosomes than mice during the early phase of the infection; this may be one of the main reasons *M. fortis* is not permissive to *S. japonicum* infection.

The changes in the splenic T lymphocyte subsets in *M. fortis*, rats and mice seen 10 days post-schistosome infection were compared in this study using FCM to characterize the cellular immune function. T cell subsets are important for the host's immune defense against infection. CD4^+^ T cells can regulate the activity of the immune response, determining B cell antibody class switching and secreting cytokines. CD8^+^ T cells are essential in immune suppression and cell toxicity. Alterations in the CD4^+^ and CD8^+^ T cell ratio resulting from a decrease or increase in CD4^+^ or CD8^+^ T cells number could lead to defects in immune function. The dynamic equilibrium of CD4^+^ and CD8^+^ T cell ratio determines the immune status and immune competence of the host, and a decrease of the CD4/CD8 ratio is generally observed in response to a variety of diseases in humans and animals [16]. Caldas et al. reported that elevated numbers of both CD4^+^ and CD8^+^ T cells and a reduction in the CD4^+^/CD8^+^ T cell ratio was observed in patients with acute schistosomiasis, this is in agreement with our results from the susceptible host, mice [17]. However, the non-susceptible host, the rat, displayed a significant increase in the percentage of CD4^+^ T cells and a significant reduction in the percent of CD8^+^ T cells, as well as an increase in the CD4^+^/CD8^+^ T cell ratio, in infected rats compared to uninfected animals. These results suggested that the changing of CD4^+^/CD8^+^ T cell ratio might impact susceptibility to *S. japonicum* infection. Furthermore, since lacking a specific T cell monoclonal antibody against *M. fortis*, we tried to analyze T cell subsets in the tissue from these animals using fluorescently-conjugated anti-rat monoclonal antibodies, and few fluorescent signals were detected by FCM as the difference of interspecies lead to the failure of antibody-antigen binding.

Microarray analyses revealed that, 10 days post-infection with *S. japonicum*, the gene expression profiles in the lungs of the non-susceptible hosts, *M. fortis* and rats, and in the susceptible host, mice, were different from the uninfected animals; this was consistent with our previous microarray results between uninfected M.fortis and mice and those challenged with *S. japonicum* for 7 d [18]. The identification of the genes changed in the lungs of *S. japonicum*-infected *M.fortis* provided information of the biological processes and biochemical pathways that were different in three species lungs. Interestingly, compared with the infected mice, there were a larger number of immune-associated genes upregulated in *M.fortis*, including *Ptprc*, *Tnfr1*, *C1qa*, *C8a*, C*gr1* and *Cgr3*, which may play an important role in immune response against schistosomes. The further analyses showed the response mechanisms to schistosome infection including members of the Jak-STAT and Notch signaling pathways. The Jak-STAT pathway is responsible for numerous physiological responses including hematopoieis, immune responses and development in mammals [19], [20]. The pathway is known to be activated by a large number of cytokines factors and plays an essential role in their signal transduction (*Jak1*, *Jak2*, *Stat*, *Grb2*, *Ptpn6*, *Myc*, *Socs2*, etc.). In more recent years, investigators found increasing evidence that Notch plays important roles during T cell-mediated immune responses, in particular for the regulation of T helper cell differentiation (*Psen1*, *Adam17*, *Hdac1*, *Ncstn*, etc.) [21], [22]. In the liver of the infected *M. fortis*, many nonspecific and specific immunity-related gene were also upregulated. Finding known, well-characterized genes, such as *Paf*, *Cd74*, *Il3*, *Ly6e*, *Irf7*, etc.Our observations in the current study suggests that the significant differences in immunopotency among the three host species may be closely related to the differential expression of the above mentioned immune-associated genes. Remarkably, after infected with schistosomes, three immune-associated genes, cathepsin S (*Ctss*), chemokine receptor-NOT transcription complex 2 (*Cnot2*) and integrin β2 (*itgb2*) are apparently up-regulated in both the lungs and liver of non-schistosome susceptible hosts, *M. fortis* and rats; they are significantly upregulated in the lungs of these hosts. In contrast, there is almost no change in the lungs of schistosome-susceptible mice. For example, *Ctss* is one type of aminothiopropionic acid cathepsin that is mainly expressed in antigen presenting cell participated the positive selection of CD4^+^ T cells and MHC class II-mediated antigen presentation through both degradation of the endocytic protein antigen and the invariant chain processing pathway [23], [24]. *Cnot2* involves in the migration of lymph cells [25]. *Itgb2* is only expressed in leukocytes, where it functions in the killing activity of cytotoxic T cells, natural killer cells and lymphokine-activated killer cells [26]. Based on our results, the upregulation of the expression of some non-specific immune-related genes in both *M. fortis* and rats infected with *S. japonicum* may also be another mechanism by which the non-susceptible hosts impacts the development, maturation and survival of *S. japonicum*; this may also be one of the underlying molecular mechanisms mediating the natural resistance displayed by *M. fortis* to *S. japonicum* infection.

In addition, in the tissue from *M.fortis* infected with *S. japonicum* for 10 days, a class of apoptosis-promoting genes were upregulated, including programmed cell death 6 (*Pdcd6*), caspase 7 (*Casp7*) and tyrosine protein kinase 2 (*Tyk2*). In general, these molecules recognize the programmed apoptosis protein receptors presented on the surface of aging or pathological cells, activate a series of intracellular signal transduction pathways and eventually lead to cell death. Jia et al. presumed that the role of these proteins in *M. fortis* might be to initiate the programmed cell death of the schistosome [27].

Other novel findings of our study included development-associated genes expressed in the lungs or liver of *M. fortis* infected with *S. japonicum* that were significantly downregulated, including *Thra*, *Thrsp*, *Hsd11b1* and *Igf1*. The studies published by Hu et al. on the *S. japonicum* genome indicated the schistosomes need to use hormones and growth factors from hosts to support and promote their own growth and development during the parasitic process [28]. The downregulation of these development-associated genes in *M. fortis* may be an important reason why *S. japonicum* cannot survive or mature in this species. The thyroid hormone receptor alpha (THRα) and thyroid hormone responsive protein (THRSP) have important roles in improving the metabolic rate, increasing oxygen consumption and promoting growth and development. Saule et al. found that mice with hypothyroidism, infected with *S. mansoni*, had reduced parasite maturation, in spite of sex differentiation, and the worms became smaller [29]. Conversely, mice infected with *S. mansoni* and treated with thyroxine had increased worm numbers, and the worms grew larger, matured earlier and laid more eggs. Brigg's study indicated that *S. mansoni* could make use of steroids from hosts and converted them into metabolic products [30]. The number of eggs laid by schistosomes could be affected when steroidal hormones are inhibited. Torpier et al. found that a high concentration of free steroid aggregated the lung-stage schistosomulum and polarity of the ecdysone steroid hormone in *S. mansoni*, mainly around the vitelline gland. Changes in steroid concentration throughout the parasite life cycle may be the result of biosynthesis or metabolism of absorbed or stored hormone. The biosynthesis of steroids has not been detected in schistosomes in vivo, but *S. mansoni* has a steroid-molting polarity connection. Insulin-like growth factor 1, also known as growth hormone stimulin C, is an important regulatory protein in cell growth and differentiation. Hu et al. found that *S. japonicum* shares some sequences with humans and mice for the insulin receptor or insulin-like growth factor 1 receptor and inferred that *S. japonicum* could accept host hormone signals for the induction of cell proliferation and development [28]. The downregulated genes, including *Thra*, *Thrsp* and *Hsd11b1* in the lungs and *Igf1* in liver of the *M.fortis* infected with schistosomes for 10 days, demonstrated that thyroid hormone does not simply influence the development of schistome through the metabolic process but it may also combine with steroids, insulin or other molecules to form an environment suitable for the development and survival of schistosomes. Thus, the role of some of these development-associated genes may be indirect. Therefore, the growth inhibition of *S. japonicum* in *M. fortis* may be related to not only the upregulation of some specific or non-specific immune-related genes, but also to the downregulation of some molecules to attempt to prevent *S. japonicum* from using proteins needed for growth and development that originate in its host.

These findings will be helpful in elucidating the key molecules active against *S. japonicum* in the *M. fortis* and for understanding more about the relative mechanism of *S. japonicum* infection and about the interaction between *S. japonicum* and their hosts. These research efforts should be intensified in the future to confirm the biological function of the differentially expressed genes in the hosts and to learn more about the molecular mechanisms of schistosome infection.

## Materials and Methods

### Animal and cercariae infection

Eight weeks old of SPF (Specific pathogen free) *M. fortis* (male, 60 g) were purchased from Shanghai Xipu'er-bikai Experimental Animal Co., Ltd. (Shanghai). SPF Wistar rats (8 weeks, male, 150 g) and BALB/c mice (8 weeks, male, 20 g) were purchased from the Shanghai laboratory animal center at the Chinese Academy of Science (Shanghai). They were raised in a sterilized room and feed sterilized food and water. *S. japonicum* cercariae (Chinese Strain) were obtained from the snail-maintaining room at the Shanghai Veterinary Research Institute, Chinese Academy of Agricultural Sciences. Twelve of each rodent species were randomly divided into four groups of three for each species. Each of the Mf, rat and mouse in group A was challenged with 3000, 2000 and 200 cercariae of *S. japonicum* respectively, and the animals in group B were regarded as the uninfected control. The Infection experiment was repeated once with group C (infected) and group D (control). All animal care and procedures were conducted according to the guidelines for animal use in toxicology. The study protocol was approved by the Animal Care and Use Committee of the Shanghai Veterinary Research Institute, Chinese Academy of Agricultural Sciences.

### Collection of tissue samples and preparation of paraffin sections

The animals were sacrificed 10 days post-infection and the lungs, liver and spleen were harvested. The lungs and liver were cut into 1.5 cm×1.5 cm×0.3 cm pieces for sectioning and the remainder of the tissue was preserved in liquid nitrogen for RNA isolation. The pieces were fixed in 10% formalin after washing with PBS. Then, the formalin-fixed samples were dehydrated in ethanol, cleared with xylene and embedded in paraffin wax. Sections (6-µm thick) were stained with hematoxylin and eosin and observed using a light microscope. The spleens were excised aseptically and preserved in PBS for analysis of the T cell subsets.

### Detection and analysis of spleen T lymphocyte subsets

The splenocytes of six *M.fortis*, six rats, and six mice were mechanically fragmented and placed in a petri dish containing 5 mL RPMI1640 medium supplemented with 10% fetal calf serum. The cell suspension was filtered through a 200 µm mesh stainless steel filter. To remove red blood cells, the pellets were resuspended in 3 mL TRIS ammonium chloride (0.144 M NH4Cl, 0.017 M TRIS, pH 7.2) and washed twice [31]. Vital cells were counted by means of trypan blue dye exclusion staining and adjusted to 5×10^6^ cells/mL in fluorescence-activated cell sorter (FACS) buffer (PBS, 1% FCS, 0.1% sodium azide, 5% normal mouse serum). The procedure for flow cytometry (FCM) staining has been described [32]. Briefly, the rats and *M. fortis* splenocytes were stained with fluorescein (FITC)-conjugated anti-rat CD4 and phycoerythrin (PE)-conjugated anti-rat CD8 and mouse splenocytes were stained with FITC-conjugated anti-mouse CD4 and PE-conjugated anti-mouse CD8. The cells were examined using a FACSCalibur flow cytometer (Becton Dickinson, San Josè, CA). FCM data analyses were performed using CellQuest software and different data management procedures were applied depending on the sample staining. Paired samples *t*-test was used to examine differences in values between different groups, and values in the text are mean ± Std. A value of *p*<0.05 was regarded as significant difference.

### Isolation of total RNA and RNA fluorescent labeling

Total RNA was isolated from the livers and lungs of each experimental animal using the TRIzol Reagent (Invitrogen). The integrity and concentration of RNA were evaluated by formaldehyde gel electrophoresis and ultraviolet spectrophotometry, respectively. For the synthesis of the cDNA by reverse transcription, 5 µg total RNA was used as the template. The cDNA was further amplified and cRNA was synthesized in vitro. The cDNA probes from the tissue of control group were labeled with dCTP-Cy5, while those from the infected mice group were labeled with dCTP-Cy3.

### Microarray hybridization and data analysis

Microarray analysis was carried out by Capital Bio, Ltd., Beijing, China. The mouse oligonucleotide arrays applied in this study contained 35,825 probe sets, representing 25,051 different genes, and the rat oligonucleotide arrays contained 26,962 probe sets, representing 22,012 different genes. The labeled cDNA was dissolved in 80 µL hybridization solution (3× SSC, 0.2% SDS, 5× Denhart's and 25% formamide) at 42°C overnight. The hybridized chips were washed at 42°C for 5 min with 2× SSC containing 0.2% SDS and then washed with 0.2× SSC at room temperature for 5 min, before being dried at room temperature for scanning. All reagents used in this procedure were provided in the Chip Hybridization Kit purchased from the Capital Bio corporation. The chips were scanned using a LuxScan 10K/A dual channel laser scanner (Capital Bio) and each was technically repeated by fluorescence exchange. The acquired images were analyzed using LuxScan 3.0 (CapitalBio), then, the overall data was normalized using Lowess regression. Every gene had a three-repeated ratio and was analyzed using the SAM software [33]. The false discovery rate (FDR) was controlled under 5%. The differentially expressed genes were defined by an increase or decrease two-times the control level as upregulation or downregulation, respectively, and analyzed using the CLUSTER and TREEVIEW software.

### Database Accession Numbers

Microarray data was generated in conformity to MIAME guidelines and has been deposited in the GEO database under accession number GSE21703.

### Real-Time RT-PCR analysis

Six genes from the different categories of differentially expressed genes were chosen to further verify the reliability of the chip results by real time RT-PCR using the DNA-binding dye SYBR green I according to the manufacturer's instructions. PCR primers were designed using sequences that were conserved among the three species and primers for β-actin were used as an internal control for normalization (see Table 4). We isolated the RNA samples from six of each infected and uninfected rodent species respectively, and the genes of interest and the housekeeping control β-actin from each sample were amplified for RT-PCR analysis in triplicate in this test. The thermal cycling protocol involved 2 minutes at 95°C, followed by 40 cycles at 95°C for 10 s, 55°C for 5 s and 72°C for 15 s. Relative quantification of gene expression was calculated as the infected sample divided by the non-infected sample 2^−ΔΔCT^ after normalization against expression. The analysis of real-time PCR was performed using the Rotor-Gene 3000A Dual Channel Multiplexing System (Corbett Research), and PCR amplifications were confirmed by electrophoresis. The result of the real-time RT-PCR was quantitatively analyzed using the comparative threshold method.

**Table 4:** Information on the primers used for quantitative real-time PCR.

	Rat			Mouse			
Gene symbol	Primer sequence (5′→3′)	Amplicon length	GenBank Accession No.	Primer sequence (5′→3′)	Amplicon length	GenBank Accession No.	Identity (%) Rat/mouse
*Actb*	CCACACCCGCCACCAGTTC GACCCATACCCACCATCACACC	167	CF111145	GAGGGAAATCGTGCGTGACATC GAACCGCTCGTTGCCAATAGTG	151	AC157380	
*Psmb8*	ACGGTTGGGTGAAAGTGGAGAG AGGGCTGGCTGAGTGAGAGG	230	NM_080767	CACAACCACACTCGCCTTCAAG GCCAACAGCCTCTCCCAGTAC	180	BC051450	90
*Cnot2*	CCCTGGGCTTCTTCACCTTGTC CCGCTCCTCCTTGTGGTATCTC	225	XM_576225	CACAAGGAGGAGCGGGTATGG TTGAAGGTGGATGGCAGGTGAG	182	BC090624	97
*C1r*	AGGTTTGTCCGTCTGCCCATAG ATCCCTCACCACACCCAATGC	229	XM_242644	CACCCAGACTACCGCCAAGATG TCCCGAATCCGCTGACATAACC	166	AF459008S4	91
*Prpf4b*	GCAGTGCTGGGTGGTTGATTTG AGGCAGGCTCAAGAAGGAACAG	180	XM_574012	TTGGTGTAGTCAGTGCCGAAGG CTATGCCACAGTTGCGATGCG	247	AF283466	96
*Fxyd1*	ATTACCACACCCTGCGGATCG GGTGGACAGACGGCGGATG	173	CK476741	ATTACCACACCCTGCGGATCG GGATGACAGACGGCGGATGG	173	BC024671	92
*Nudt18*	CCTGCCCACACCGCTTAGAG ACCCACCAACACCCACACTG	175	XM_341351	CCTGCCCACACCGCTTAGAG ACCGACTGGACTGTTGTGAAGG	159	AC122268	93

## Supporting Information

Table S1 Differential expressed genes in lungs and livers of three species with *S. japonicum* infection. Ratio of expression level calculated by the infected sample divided by the noninfected sample.
(0.20 MB DOC)

Table S2 All the changed expression genes in the three species.
(2.26 MB XLS)
