# Supplementary material for: Study on Differences in the Pathology, T Cell Subsets and Gene Expression in Susceptible and Non-Susceptible Hosts Infected with Schistosoma japonicum
Source: PLoS One. 2010 Oct 18;5(10):e13494. doi: 10.1371/journal.pone.0013494 (PMC2956682; doi:10.1371/journal.pone.0013494)
Supplement: Table S1 — Differential expressed genes in lungs and livers of three species with S. japonicum infection. Ratio of expression level calculated by the infected sample divided by the noninfected sample. (0.20 MB DOC) [file pone.0013494.s001.doc]

| **Table. 4 Differential expressed genes in lungs and livers of three species with *S. japonicum* infection**  **(Ratio of expression level calculated by the infected sample divided by the noninfected sample).** | | | | | | | | | |
| --- | --- | --- | --- | --- | --- | --- | --- | --- | --- |
| name | GenBank accession number | | Annotation | lung | | | liver | | |
|  | rat | mouse |  | *Mf* | rat | mouse | *Mf* | rat | mouse |
| Immunoglobulins/related genes | | | | | | | | | |
| Itgb2 | BC085817 | X14951 | integrin beta 2 | 12.30 | 2.19 | 1.48 | 2.12 | 2.20 | 1.15 |
| Arl6ip2 | XM_216629 | AF133670 | ADP-ribosylation factor-like 6 interacting protein 2 | 5.52 | 1.74 | 1.37 | 2.29 | 1.53 | 0.92 |
| C1r | XM_242644 | AF459008S4 | complement component 1, r subcomponent | 3.74 | 1.33 | 1.03 | 2.43 | 4.33 | 0.97 |
| Lsp1 | XM_341964 | MUSS37X | lymphocyte specific 1 | 3.38 | 2.04 | 1.02 | 3.15 | 1.56 | 0.73 |
| C1qa | BC086605 | BC002086 | complement component 1, q subcomponent, alpha polypeptide | 2.85 | 1.69 | 1.42 | 2.18 | 0.65 | 1.11 |
| Psmb8 | NM_080767 | BC051450 | proteosome subunit, beta type 8 | 2.08 | 2.31 | 1.03 | 4.31 | 2.92 | 1.24 |
| Signal transduction | | | | | | | | | |
| Msr1 | XM_573919 | AC111028 | macrophage scavenger receptor 1 | 7.77 | 2.18 | 1.34 | 2.32 | 1.21 | 0.88 |
| Pnrc1 | U61729 | AL670464 | proline-rich nuclear receptor coactivator 1 | 4.25 | 2.29 | 1.17 | 2.37 | 2.52 | 1.18 |
| Rgs7bp | XM_574845 | AC154297 | regulator of G-protein signalling 7 binding protein | 0.13 | 0.24 | 0.68 | 0.29 | 0.56 | 0.6 |
| Gene transcriptional regulation | | | | | | | | | |
| Pnrc2 | BC081903 | BC006598 | proline-rich nuclear receptor coactivator 2 | 12.21 | 2.48 | 1.15 | 2.48 | 2.43 | 0.83 |
| Cnot2 | XM_576225 | BC090624 | CCR4-NOT transcription complex, subunit 2 | 9.89 | 2.93 | 0.93 | 3.57 | 4.57 | 1.11 |
| Gpbp1 | XM_215485 | AC118475 | GC-rich promoter binding protein 1 | 7.67 | 4.92 | 1.19 | 2.58 | 6.92 | 0.96 |
| Mll3 | DN936424 | AC127319 | myeloid/lymphoid or mixed-lineage leukemia 3 | 6.49 | 1.98 | 0.84 | 2.45 | 1.24 | 0.99 |
| Rbm39 | BC078917 | BC086645 | RNA binding motif protein 39 | 4.37 | 0.65 | 1.18 | 2.01 | 2.97 | 0.84 |
| Xbp1 | BC079450 | BC029197 | X-box binding protein 1 | 2.63 | 0.65 | 0.98 | 2.20 | 2.33 | 0.96 |
| Ewsr1 | BC098822 | X79233 | Ewing sarcoma breakpoint region 1 | 2.01 | 0.94 | 0.89 | 2.10 | 1.19 | 0.66 |
| Znrd1 | NM_213567 | AF230340 | zinc ribbon domain containing, 1 | 0.36 | 1.68 | 0.99 | 0.49 | 0.56 | 0.86 |
| Cited2 | NM_053698 | Y15163 | Cbp/p300-interacting transactivator, with Glu/Asp-rich carboxy-terminal domain, 2 | 0.28 | 0.54 | 0.86 | 0.50 | 0.77 | 1.02 |
| Transport | | | | | | | | | |
| Gdi2 | NM_017276 | AF095728 | guanosine diphosphate (GDP) dissociation inhibitor 2 | 10.39 | 4.40 | 1.05 | 3.35 | 5.21 | 1.00 |
| Slc25a4 | BG670788 | X74510 | solute carrier family 25 member 4 | 2.15 | 1.24 | 1.07 | 3.09 | 1.34 | 0.79 |
| Scarb1 | AB002151 | MMU37799 | scavenger receptor class B, member 1 | 0.50 | 0.77 | 0.93 | 0.44 | 1.42 | 1.25 |
| Ndufa2 | AI103694 | AF124786 | NADH dehydrogenase 1 alpha subcomplex, 2 | 0.41 | 2.06 | 1.13 | 0.45 | 0.56 | 0.98 |
| Tst | CO404031 | MMU35741 | thiosulfate sulfurtransferase, mitochondrial | 0.41 | 1.72 | 0.94 | 0.50 | 0.8 | 0.86 |
| Tmem112 | XM_340769 | BC020104 | transmembrane protein 112 | 0.34 | 0.32 | 0.93 | 0.24 | 0.56 | 1.33 |
| Fxyd1 | CK476741 | AF091390 | FXYD domain-containing ion transport regulator 1 | 0.33 | 1.62 | 0.89 | 0.40 | 0.76 | 1.13 |
| Nfic | CK839915 | Y07692 | nuclear factor I/C | 0.19 | 0.55 | 1.06 | 0.50 | 1.05 | 1.92 |
| Ndufb7 | BG668391 | BC056984 | NADH dehydrogenase 1 beta subcomplex, 7 | 0.19 | 0.94 | 0.87 | 0.49 | 0.51 | 0.90 |
| Apoptosis | | | | | | | | | |
| Dap3 | BC085878 | AJ250375 | death associated protein 3 | 0.38 | 1.33 | 0.95 | 0.49 | 0.82 | 0.92 |
| Fkbp8 | BC107454 | AF030635 | FK506 binding protein 8 | 0.38 | 0.92 | 0.80 | 0.30 | 0.80 | 1.29 |
| Enzyme activity | | | | | | | | | |
| Ctss | BC059142 | BC011104 | cathepsin S | 17.89 | 2.32 | 1.59 | 3.16 | 2.97 | 0.86 |
| Stt3b | XM_343499 | BC052433 | STT3, subunit of the oligosaccharyl transferase complex, homolog B | 7.15 | 2.49 | 1.25 | 2.02 | 2.33 | 1.19 |
| Clk1 | BC081942 | X57186 | CDC-like kinase 1 | 2.86 | 1.85 | 1.31 | 3.71 | 2.29 | 0.82 |
| 2610507B11Rik | XM_220636 | AK122206 | RIKEN cDNA 2610507B11 gene | 2.00 | 0.82 | 1.16 | 2.63 | 1.82 | 1.14 |
| Tbc1d2 | XM_232990 | XM_283964 | TBC1 domain family, member 2 | 0.50 | 1.46 | 1.16 | 0.47 | 0.67 | 0.95 |
| Pck2 | XM_341319 | BC023437 | phosphoenolpyruvate carboxykinase 2 | 0.42 | 0.90 | 0.99 | 0.49 | 0.82 | 0.86 |
| Dhrs7b | BC086453 | AC096627 | dehydrogenase/reductase member 7B | 0.42 | 1.58 | 0.98 | 0.25 | 0.56 | 0.96 |
| Nudt18 | XM_341351 | AC122268 | nudix type motif 18 | 0.39 | 0.75 | 1.01 | 0.42 | 0.78 | 1.36 |
| Abhd6 | BC083826 | BC027011 | abhydrolase domain containing 6 | 0.28 | 0.35 | 1.17 | 0.43 | 0.72 | 1.07 |
| Enzymes | | | | | | | | | |
| Cmpk | BC098727 | BC017684 | cytidylate kinase | 15.98 | 1.02 | 1.27 | 2.07 | 3.63 | 0.94 |
| Csnk1a1 | CF110397 | X90945 | casein kinase 1, alpha 1 | 3.81 | 1.64 | 1.13 | 2.08 | 1.40 | 1.25 |
| Acot7 | BC085900 | AL772240 | acyl-CoA thioesterase 7 | 0.31 | 1.41 | 1.02 | 0.49 | 0.34 | 0.83 |
| Metabolism | | | | | | | | | |
| Hmgcr | NM_013134 | BC085083 | 3-hydroxy-3-methylglutaryl-Coenzyme A reductase | 3.28 | 2.77 | 0.89 | 3.79 | 2.52 | 0.84 |
| Idh1 | CO563533 | AC101869 | isocitrate dehydrogenase 1 (NADP+), soluble | 7.83 | 1.69 | 1.27 | 2.95 | 5.77 | 1.06 |
| Growth | | | | | | | | | |
| Picalm | AY724477 | BC057683 | Phosphatidylinositol binding clathrin assembly protein | 21.24 | 2.42 | 1.00 | 3.41 | 3.66 | 1.28 |
| Vegfa | AY033508 | BC061468 | vascular endothelial growth factor A | 2.58 | 1.38 | 0.93 | 2.10 | 1.43 | 1.10 |
| Gadd45gip1 | CO383247 | BC061069 | growth arrest and DNA-damage-inducible, gamma interacting protein 1 | 0.33 | 1.14 | 0.91 | 0.48 | 0.83 | 1.36 |
| Protein binding | | | | | | | | | |
| Sf3b1 | XM_343570 | AB037890 | splicing factor 3b, subunit 1 | 4.60 | 1.84 | 0.84 | 2.25 | 1.98 | 1.28 |
| Sdc4 | NM_012649 | D89572 | syndecan 4 | 4.47 | 0.89 | 1.02 | 3.43 | 3.23 | 1.04 |
| Dars | BC072534 | AC161170 | aspartyl-tRNA synthetase | 2.65 | 1.81 | 0.94 | 2.03 | 2.16 | 0.76 |
| Lrrc57 | BC089966 | BC034894 | leucine rich repeat containing 57 | 2.36 | 0.75 | 1.15 | 2.08 | 1.79 | 0.85 |
| Ap2s1 | CO564866 | AL954336 | adaptor-related protein complex 2, sigma 1 subunit | 0.39 | 0.58 | 0.85 | 0.50 | 0.49 | 0.86 |
| Cell differentiation and development | | | | | | | | | |
| Fn1 | X15906 | BC036167 | fibronectin 1 | 5.70 | 2.84 | 0.98 | 2.72 | 3.64 | 1.39 |
| Birc6 | XM_233842 | Y17267 | baculoviral IAP repeat-containing 6 | 4.78 | 0.79 | 1.01 | 3.15 | 2.37 | 0.65 |
| Plscr1 | BF403965 | D78355 | phospholipid scramblase 1 | 3.50 | 1.14 | 1.07 | 2.23 | 1.51 | 1.01 |
| Nucleic acid binding | | | | | | | | | |
| Arf4 | BC063167 | D87901 | ADP-ribosylation factor 4 | 9.19 | 1.29 | 1.15 | 2.41 | 5.10 | 0.75 |
| Zfp281 | BC083844 | AC125186 | zinc finger protein 281 | 4.55 | 1.04 | 0.99 | 2.55 | 1.61 | 1.04 |
| Ints1 | XM_213717 | BC063266 | integrator complex subunit 1 | 0.48 | 0.86 | 0.96 | 0.16 | 0.92 | 1.06 |
| Nucb1 | BC100643 | BC072554 | nucleobindin 1 | 0.46 | 0.72 | 0.9 | 0.34 | 1.24 | 1.23 |
| Hist1h2ao | CB581786 | AY158913 | histone cluster 2, H3c2 | 0.38 | 0.6 | 1.01 | 0.42 | 0.62 | 1.30 |
| RNA processing | | | | | | | | | |
| Hnrph1 | BC099792 | BC056224 | heterogeneous nuclear ribonucleoprotein H1 | 8.34 | 1.38 | 0.94 | 2.42 | 1.22 | 0.74 |
| Dhx15 | XM_214053 | AC102739 | DEAH (Asp-Glu-Ala-His) box polypeptide 15 | 7.69 | 1.06 | 1.07 | 2.39 | 1.71 | 0.87 |
| Prpf4b | CO403277 | MMU48737 | PRP4 pre-mRNA processing factor 4 homolog B | 5.10 | 1.88 | 1.03 | 2.29 | 1.91 | 0.70 |
| Ddx5 | BC079036 | X65627 | DEAD (Asp-Glu-Ala-Asp) box polypeptide 5 | 4.77 | 0.78 | 0.80 | 2.24 | 2.45 | 0.79 |
| Cell surface markers and membrane proteins | | | | | | | | | |
| Cd9 | CF111078 | AC153580 | CD9 antigen | 31.69 | 2.08 | 1.09 | 3.92 | 2.17 | 0.84 |
| Stress response | | | | | | | | | |
| Xpa | XM_216403 | X74351; | xeroderma pigmentosum, complementation group A | 0.40 | 1.31 | 0.92 | 0.48 | 0.99 | 0.88 |
| Cell adhesion | | | | | | | | | |
| Sympk | XM_214843 | BC049852 | symplekin | 0.49 | 0.95 | 1.04 | 0.48 | 0.77 | 1.50 |
| Unclassified | | | | | | | | | |
| 1110002B05Rik | XM_580102 | BC022674 | RIKEN cDNA 1110002B05 gene | 11.96 | 1.76 | 1.07 | 2.39 | 4.62 | 1.05 |
| BC005537 | BC085940 | BC005537 | cDNA sequence BC005537 | 6.23 | 0.83 | 1.42 | 2.63 | 2.7 | 1.52 |
| 1600014C10Rik | CB739877 | BC085480 | RIKEN cDNA 1600014C10 gene | 5.57 | 1.03 | 1.35 | 2.46 | 3.39 | 1.03 |
| Fam18b | XM_219680 | BC115505; | family with sequence similarity 18, member B | 5.24 | 2.05 | 1.30 | 2.56 | 1.31 | 0.80 |
| Prg1 | NM_020074 | BC037076 | proteoglycan 1, secretory granule | 4.47 | 2.13 | 1.18 | 3.15 | 1.12 | 0.86 |
| BC008155 | BC104693 | AC154589 | cDNA sequence BC008155 | 0.44 | 1.85 | 0.98 | 0.48 | 0.67 | 0.96 |
| 1110008P14Rik | XM_575113 | BC049639 | RIKEN cDNA 1110008P14 gene | 0.44 | 1.08 | 0.83 | 0.44 | 0.58 | 1.12 |
| 4933404M19Rik | BC098036 | AC004807 | RIKEN cDNA 4933404M19 gene | 0.27 | 0.28 | 0.93 | 0.16 | 0.35 | 1.33 |
